# Author Correction: *Daphnia* stressor database: Taking advantage of a decade of *Daphnia* ‘-omics’ data for gene annotation

**DOI:** 10.1038/s41598-020-60907-5

**Published:** 2020-03-03

**Authors:** Suda Parimala Ravindran, Jennifer Lüneburg, Lisa Gottschlich, Verena Tams, Mathilde Cordellier

**Affiliations:** 1 Department of Marine Sciences, Tjärnö Marine Laboratory, University of Gothenburg, 452 96 Strömstad, Sweden; 2 Universität Hamburg, Institute of Zoology, Martin-Luther-King Platz 3, 20146 Hamburg, Germany; 3 Universität Hamburg, Institut für marine Ökosystem- und Fischereiwissenschaften, Große Elbstraße 133, 22767 Hamburg, Germany

Correction to: *Scientific Reports* 10.1038/s41598-019-47226-0, published online 31 July 2019

In the original version of this Article, Jennifer Lüneburg and Lisa Gottschlich were incorrectly affiliated with ‘Universität Hamburg, Institut für marine Ökosystem- und Fischereiwissenschaften, Große Elbstraße 133, 22767 Hamburg, Germany’. In addition, Verena Tams was incorrectly affiliated with ‘Universität Hamburg, Institute of Zoology, Martin-Luther-King Platz 3, 20146, Hamburg, Germany’.

The correct affiliations are listed below:

Affiliation 1:

Department of Marine Sciences, Tjärnö Marine Laboratory, University of Gothenburg, 452 96 Strömstad, Sweden

Suda Parimala Ravindran

Affiliation 2:

Universität Hamburg, Institute of Zoology, Martin-Luther-King Platz 3, 20146 Hamburg, Germany

Jennifer Lüneburg, Lisa Gottschlich, Mathilde Cordellier

Affiliation 3:

Universität Hamburg, Institut für marine Ökosystem- und Fischereiwissenschaften, Große Elbstraße 133, 22767 Hamburg, Germany

Verena Tams

This error has now been corrected in the PDF and HTML versions and Supplementary Information file which accompanies the Article.

