# Supplementary material for: Daphnia stressor database: Taking advantage of a decade of Daphnia ‘-omics’ data for gene annotation
Source: Sci Rep. 2019 Jul 31;9:11135. doi: 10.1038/s41598-019-47226-0 (PMC6668405; doi:10.1038/s41598-019-47226-0)
Supplement: Supplementary file 1 — List of gene expression studies in Daphnia [file 41598_2019_47226_MOESM1_ESM.pdf]

*Daphnia s*tressor database: Taking advantage of a decade of *Daphnia* '-omics' data for gene annotation

Suda Parimala Ravindran[1], Jennifer Lüneburg[2], Lisa Gottschlich[2], Verena Tams[2,3] and Mathilde Cordellier[2],*

Supporting information File 1:

Table S1: List of gene expression studies in *Daphnia*, authors of the paper, year of publication, stressors used, species, experimental technique used in the study.

Title	Authors	Publicatio	Technique used	Species	Stressor(s) used in the study
Acclimatory responses of the Daphnia pulex proteome to environmental changes. II. Chronic exposure to different temperatures (10 and 20 degrees C) mainly affects protein metabolism	Schwerin, S., Zeis, B., Lamkemeyer, T., Paul, R. J., Koch, M., Madlung, J., Fladerer, C. and Pirow, R.	2009	Protein 2D-Gel Electrophoresis	Daphnia pulex	Temperature
Adjustments of serine proteases of Daphnia pulex in response to temperature changes	Dolling, R., Becker, D., Hawat, S., Koch, M., Schwarzenberger, A. and Zeis, B.	2016	qPCR	Daphnia pulex	temperature
Assessment of chemical mixtures and groundwater effects on Daphnia magna transcriptomics	Garcia-Reyero, N., Escalon, B. L., Loh, P. R., Laird, J. G., Kennedy, A. J., Berger, B. and Perkins, E. J.	2012	qPCR, microarray	Daphnia magna	RDX; 2; 6-DNT; Tri Nitro Benzene; MIX-1; MIX-3; MIX-4; MIX-5; MIX-6; MIX-7; MIX-8; LAAP-85; LAAP-108; Tri Nitro Toluene; 2; 4-DNT; Dinitrobenzene; MIX-2
Biomarker Discovery and Transcriptomic Responses in Daphnia magna exposed to Munitions Constituents	Garcia-Reyero, N., Poynton, H., Kennedy, A., Guan, X., Escalon, B., Chang, B., Varshavsky, J., Loguinov, A., Vulpe, C. and Perkins, E. J.	2009	microarray	Daphnia magna	Tri Nitro Benzene; Copper; Zinc; WO4; 4-ADNT; Lead; RDX; 2-ADNT; 2; 4-DNT; Dinitrobenzene; Tri Nitro Toluene; 2; 6-DNT
Can metal stress induce transferable changes in gene transcription in Daphnia magna?	Vandegehuchte, M. B., Vandenbrouck, T., De Coninck, D., De Coen, W. M. and Janssen, C. R.	2010	microarray	Daphnia magna	Zinc
Candidate innate immune system gene expression in the ecological model Daphnia	Decaestecker, E., Labbe, P., Ellegaard, K., Allen, J. E. and Little, T. J.	2011	qPCR	Daphnia magna	Pasteuria ramosa
Chronic toxicity evaluation of the flame retardant tris (2-butoxyethyl) phosphate (TBOEP) using Daphnia magna transcriptomic response	Giraudo, M., Douville, M. and Houde, M.	2015	microarray	Daphnia magna	Tris (2-butoxyethyl) phosphate (TBOEP)
Cloning, expression and localization of DacaCSP2 and DacaCSP3 during different reproductive stages in Daphnia carinata	Li, H., Yang, Y., Xu, G., Wu, D., Lv, W., Jiang, Q. and Zhao, Y.	2016	qPCR	Daphnia carinata	Reproductive stages; Temperature
Cloning, expression and localization of the Daphnia carinata transformer gene DcarTra during different reproductive stages	Kong, L., Lv, W., Huang, Y., Liu, Z., Yang, Y. and Zhao, Y.	2015	qPCR	Daphnia carinata	Reproductive stages
Comparative ovarian microarray analysis of juvenile hormone-responsive genes in water flea Daphnia magna: potential targets for toxicity	Toyota, K., Williams, T. D., Sato, T., Tatarazako, N. and Iguchi, T.	2017	microarray	Daphnia magna	Fenoxycarb; methyl farnesoate
Conserved Transcription Factors Steer Growth-Related Genomic Programs in Daphnia	Spanier, K. I., Jansen, M., Decaestecker, E., Hulselmans, G., Becker, D., Colbourne, J. K., Orsini, L., De Meester, L. and Aerts, S.	2017	RNA-Seq	Daphnia pulex	Temperature
Conserved transcriptional responses to cyanobacterial stressors are mediated by alternate regulation of paralogous genes in Daphnia	Asselman, J., Pfrender, M. E., Lopez, J. A., De Coninck, D. I., Janssen, C. R., Shaw, J. R. and De Schamphelaere, K. A.	2015	microarray	Daphnia pulex	Cyanobacteria
Core chemistry influences the toxicity of multicomponent metal oxide nanomaterials, lithium nickel manganese cobalt oxide, and lithium cobalt oxide to Daphnia magna	Bozich, J., Hang, M., Hamers, R. and Klaper, R.	2017	qPCR	Daphnia magna	Lithium cobalt oxide; Lithium nickel manganese cobalt oxide
Daphnia magna and ecotoxicogenomics: gene expression profiles of the anti-ecdysteroidal fungicide fenarimol using energy-, molting- and life stage-related cDNA libraries	Soetaert, A., van der Ven, K., Moens, L. N., Vandenbrouck, T., van Remortel, P. and De Coen, W. M.	2007	microarray	Daphnia magna	Fenarimol
Daphnia magna Ecotoxicogenomics Provides Mechanistic Insights into Metal Toxicity	Poynton, H. C., Varshavsky, J. R., Chang, B., Cavigiolio, G., Chan, S., Holman, P. S., Loguinov, A. V., Bauer, D. J., Komachi, K., Theil, E. C., Perkins, E. J., Hughes, O. and Vulpe, C. D.	2007	microarray	Daphnia magna	Cadmium; Copper; Zinc
Deciphering mechanisms of malathion toxicity under pulse exposure of the freshwater cladoceran Daphnia magna	Trac, L. N., Andersen, O. and Palmqvist, A.	2016	qPCR	Daphnia magna	malathion
Deciphering the genetic basis of microcystin tolerance	Schwarzenberger, A., Sadler, T., Motameny, S., Ben-Khalifa, K., Frommolt, P., Altmüller, J., Konrad, K. and von Elert, E.	2014	RNA-Seq	Daphnia magna	Chlamydomonas; Microcystin
Diet quality determines lipase gene expression and lipase/esterase activity in Daphnia pulex	Koussoroplis, A. M., Schwarzenberger, A. and Wacker, A.	2017	qPCR	Daphnia pulex	Synechococcus elongatus
Differential transcriptomic responses of ancient and modern Daphnia genotypes to phosphorus supply	Roy Chowdhury, P., Frisch, D., Becker, D., Lopez, J. A., Weider, L. J., Colbourne, J. K. and Jeyasingh, P. D.	2015	microarray	Daphnia pulex	Phosphorus
Distinct expression profiles of stress defense and DNA repair genes in Daphnia pulex exposed to cadmium, zinc, and quantum dots	Tang, S., Wu, Y., Ryan, C. N., Yu, S., Qin, G., Edwards, D. S. and Mayer, G. D.	2015	qPCR	Daphnia pulex	Cadmium; zinc; quantum dots
Early transcriptional response pathways in Daphnia magna are coordinated in networks of crustacean-specific genes	Orsini, L., Brown, J. B., Shams Solari, O., Li, D., He, S., Podicheti, R., Stoiber, M. H., Spanier, K. I., Gilbert, D., Jansen, M., Rusch, D. B., Pfrender, M. E., Colbourne, J. K., Frilander, M. J., Kvist, J., Decaestecker, E., De	2017	RNA-Seq	Daphnia magna	Cadmium; Sodium Chloride; Microcystin; carbaryl; Microcystin-free cyanobacteria; Fish Kairomones; UV; pH 5.5; Lead; crowding
Ecological genomics in Daphnia: stress responses and environmental sex determination	Eads, B. D., Andrews, J. and Colbourne, J. K.	2008	microarray	Daphnia magna and D	Methyl Farnesoate

Ecotoxicogenomic approaches for understanding molecular mechanisms of environmental chemical toxicity using aquatic invertebrate, Daphnia model organism	Kim, H. J., Koedrith, P. and Seo, Y. R.	2015	Comparative genomics	Daphnia magna	Propicanazole; cadmium; copper; zinc; Ibuprofen; bisphenol-A; silver nanoparticles; Copper sulphate; Hydrogen peroxide; pentaclorophenol; ß-naphthaflavone
Effects of charge and surface ligand properties of nanoparticles on oxidative stress and gene expression within the gut of Daphnia magna	Dominguez, G. A., Lohse, S. E., Torelli, M. D., Murphy, C. J., Hamers, R. J., Orr, G. and Klaper, R. D.	2015	qPCR	Daphnia magna	Nanoparticles
Effects of environmental contaminants on hemoglobin gene expression in Daphnia magna: a potential biomarker for freshwater quality monitoring	Ha, M. H. and Choi, J.	2009	qPCR	Daphnia magna	nonylphenol; bisphenol A; Benzopyrene; Chloropyriphos; Paraquat dichloride; Lead nitrate
Effects of glyphosate and methidathion on the expression of the Dhb, Vtg, Arnt, CYP4 and CYP314 in Daphnia magna	Le, T. H., Lim, E. S., Lee, S. K., Choi, Y. W., Kim, Y. H. and Min, J.	2010	qPCR	Daphnia magna	glyphosate; methidathion; pesticides
Effects of midazolam, pentobarbital and ketamine on the mRNA expression of ion channels in a model organism Daphnia pulex	Dong, C., Hu, A., Ni, Y., Zuo, Y. and Li, G. H.	2013	qPCR	Daphnia pulex	midazolam; pentobarbital; ketamine; Flumazenil
Endocrine-disruption potential of perfluoroethylcyclohexane sulfonate (PFECHS) in chronically exposed Daphnia magna	Houde, M., Douville, M., Giraudo, M., Jean, K., Lepine, M., Spencer, C. and De Silva, A. O.	2016	qPCR, microarray	Daphnia magna	perfluoroethylcyclohexane sulfonate (PFECHS)
Ethanol alters the expression of ion channel genes in Daphnia pulex	Hu, A. M., Zhu, T., Dong, L., Luo, N. F. and Du, G. Z.	2016	qPCR	Daphnia pulex	Ethanol
Expression and ecdysteroid responsiveness of the nuclear receptors HR3 and E75 in the crustacean Daphnia magna	Hannas, B. R. and LeBlanc, G. A.	2010	qPCR	Daphnia magna	ecdysteroids
Expression of target and reference genes in Daphnia magna exposed to ibuprofen	Heckmann, L. H., Connon, R., Hutchinson, T. H., Maund, S. J., Sibly, R. M. and Callaghan, A.	2006	qPCR	Daphnia magna	ibuprofen
Functional genomics of acclimation and adaptation in response to thermal stress in Daphnia	Yampolsky, L. Y., Zeng, E., Lopez, J., Williams, P. J., Dick, K. B., Colbourne, J. K. and Pfrender, M. E.	2014	microarray	Daphnia pulex	temperature
Gene expression and activity of digestive proteases in Daphnia: effects of cyanobacterial protease inhibitors	Schwarzenberger, A., Zitt, A., Kroth, P., Mueller, S. and Von Elert, E.	2010	qPCR	Daphnia magna	cyanobacterial protease
Gene Expression Profiling in Daphnia magna Part I: Concentration-Dependent Profiles Provide Support for the No Observed Transcriptional Effect Level	Poynton, H. C., Loguinov, A. V., Varshavsky, J. R., Chan, S., Perkins, E. J. and Vulpe, C. D.	2008	qPCR, microarray	Daphnia magna	Copper; Cadmium; zinc
Gene Expression Profiling in Daphnia magna, Part II: Validation of a Copper Specific Gene Expression Signature with Effluent from Two Copper Mines in California	Poynton, H. C., Zuzow, R., Loguinov, A. V., Perkins, E. J. and Vulpe, C. D.	2008	qPCR, microarray	Daphnia magna	Copper; cadmium; zinc
Gene expression profiling of three different stressors in the water flea Daphnia magna	Jansen, M., Vergauwen, L., Vandenbrouck, T., Knapen, D., Dom, N., Spanier, K. I., Cielen, A. and De Meester, L.	2013	microarray	Daphnia magna	carbaryl; Pasteuria ramosa; fish kairomones
Gene expression under multiple stressors in Daphnia pulex	Altshuler, I.	2012	qPCR	Daphnia pulex	calcium; fish kairomones
Gene expression variation in duplicate lactate dehydrogenase genes: do ecological species show distinct responses?	Cristescu, M. E., Demiri, B., Altshuler, I. and Crease, T. J.	2014	qPCR	Daphnia pulex and Da	temperature; oxygen
Gene response of CYP360A, CYP314, and GST and whole-organism changes in Daphnia magna exposed to ibuprofen	Wang, L., Peng, Y., Nie, X., Pan, B., Ku, P. and Bao, S.	2016	qPCR	Daphnia magna	ibuprofen
Gene response profiles for Daphnia pulex exposed to the environmental stressor cadmium reveals novel crustacean metallothioneins	Shaw, J. R., Colbourne, J. K., Davey, J. C., Glaholt, S. P., Hampton, T. H., Chen, C. Y., Folt, C. L. and Hamilton, J. W.	2007	microarray	Daphnia pulex	cadmium
Gene transcription and higher-level effects of multigenerational Zn exposure in Daphnia magna	Vandegehuchte, M. B., Vandenbrouck, T., De Coninck, D., De Coen, W. M. and Janssen, C. R.	2010	microarray	Daphnia magna	Zinc
Gene transcription, metabolite and lipid profiling in eco-indicator Daphnia magna indicate diverse mechanisms of toxicity by legacy and emerging flame-retardants	Scanlan, L. D., Loguinov, A. V., Teng, Q., Antczak, P., Dailey, K. P., Nowinski, D. T., Kornbluh, J., Lin, X. X., Lachenauer, E., Arai, A., Douglas, N. K., Falciani, F., Stapleton, H. M. and Vulpe, C. D.	2015	microarray	Daphnia magna	flame-retardants (FR)
Gene up-regulation in response to predator kairomones in the water flea, Daphnia pulex	Miyakawa, H., Imai, M., Sugimoto, N., Ishikawa, Y., Ishikawa, A., Ishigaki, H., Okada, Y., Miyazaki, S., Koshikawa, S., Cornette, R. and Miura, T.	2010	RT-qPCR	Daphnia pulex	fish kairomones
Genome-wide profiling of 24 hr diel rhythmicity in the water flea, Daphnia pulex: network analysis reveals rhythmic gene expression and enhances functional gene annotation	Rund, S. S., Yoo, B., Alam, C., Green, T., Stephens, M. T., Zeng, E., George, G. F., Sheppard, A. D., Duffield, G. E., Milenkovic, T. and Pfrender, M. E.	2016	microarray	Daphnia pulex	Light Dark Cycle
Genome-wide transcription profiles reveal genotype-dependent responses of biological pathways and gene-families in Daphnia exposed to single and mixed stressors	De Coninck, D. I., Asselman, J., Glaholt, S., Janssen, C. R., Colbourne, J. K., Shaw, J. R. and De Schamphelaere, K. A.	2014	microarray	Daphnia pulex	Cadmium; Microcystis

Genomic expression responses toward bisphenol-A toxicity in Daphnia magna in terms of reproductive activity	Jeong, S. W., Lee, S. M., Yum, S. S., Iguchi, T. and Seo, Y. R.	2013	microarray	Daphnia magna	Bisphenol A (BPA)
How do consumers deal with stoichiometric constraints? Lessons from functional genomics using Daphnia pulex	Jeyasingh, P. D., Ragavendran, A., Paland, S., Lopez, J. A., Sterner, R. W. and Colbourne, J. K.	2011	microarray	Daphnia pulex	Phosphorous
Identification and expression analysis of a doublesex1 gene in Daphnia pulex during different reproductive stages	Xu, S. L., Zhou, W., Chen, P., Zhou, J. K., Zou, X., Wang, C. L., Wang, D. L. and Zhao, Y. L.	2014	qPCR	Daphnia pulex	Developmental Stages
Induction of multixenobiotic defense mechanisms in resistant Daphnia magna clones as a general cellular response to stress	Jordao, R., Campos, B., Lemos, M. F., Soares, A. M., Tauler, R. and Barata, C.	2016	qPCR	Daphnia magna	multixenobiotic
Integration of molecular with higher-level effects of dietary zinc exposure in Daphnia magna	De Schamphelaere, K. A., Vandenbrouck, T., Muyssen, B. T., Soetaert, A., Blust, R., De Coen, W. and Janssen, C. R.	2008	qPCR, microarray	Daphnia magna	Zinc
Interactions of methyl farnesoate and related compounds with a crustacean retinoid X receptor	Wang, Y. H. and LeBlanc, G. A.	2009	Assay	Daphnia magna	methyl farnesoate
Ionotropic glutamate receptors mediate inducible defense in the water flea Daphnia pulex	Miyakawa, H., Sato, M., Colbourne, J. K. and Iguchi, T.	2015	microarray	Daphnia pulex	fish kairomones
Linking Molecular and Population Stress Responses in Daphnia magna exposed to cadmium	Connon, R., Hooper, H. L., Sibly, R. M., Lim, F.-L., Heckmann, L.-H., Moore, D. J., Watanabe, H., Soetaert, A., Cook, K., Maund, S. J., Hutchinson, T. H., Moggs, J., Coen, W. D., Iguchi, T. and Callaghan, A.	2008	microarray	Daphnia magna	Cadmium
Melatonin synthesis follows a daily cycle in Daphnia	Schwarzenberger, A. and Wacker, A.	2015	qPCR	Daphnia pulex	Light Dark Cycle; Melatonin
Metallothionein and Hsp70 trade-off against one another in Daphnia magna cross-tolerance to cadmium and heat stress	Haap, T., Schwarz, S. and Kohler, H. R.	2016	qPCR	Daphnia magna	cadmium; temperature
Metformin preconditioning protects Daphnia pulex from lethal hypoxic insult involving AMPK, HIF and mTOR signaling	Sheng, B., Liu, J. and Li, G. H.	2012	qPCR	Daphnia pulex	Metformin
Molecular characterization of the gene checkpoint homolog 1 in Daphnia carinata during different reproductive phases	Kong, L., Li, H. X., Wu, D. L., Xu, G. R., Wang, D. L. and Zhao, Y. L.	2016	qPCR	Daphnia carinata	Developmental stages
Molecular cloning and sexually dimorphic expression of DM-domain genes in Daphnia magna	Kato, Y., Kobayashi, K., Oda, S., Colbourn, J. K., Tatarazako, N., Watanabe, H. and Iguchi, T.	2008	qPCR	Daphnia magna	Sex organs
Molecular cloning of manganese superoxide dismutase gene in the cladoceran Daphnia magna: effects of microcystin, nitrite, and cadmium on gene expression profiles	Lyu, K., Zhu, X., Chen, R., Chen, Y. and Yang, Z.	2014	qPCR	Daphnia magna	microcystin; nitrite; cadmium
Molecular cloning, characterization and expression analysis of a Doublesex gene from Daphnia carinata (Crustacea: Cladocera) during different reproductive stages	Zhang, M. Q., Ma, C. A., Lv, W. W., Huang, Y. H., Wang, D. L. and Zhao, Y. L.	2015	qPCR	Daphnia carinata	Reproductive stages
Molecular impact of juvenile hormone agonists on neonatal Daphnia magna	Toyota, K., Kato, Y., Miyakawa, H., Yatsu, R., Mizutani, T., Ogino, Y., Miyagawa, S., Watanabe, H., Nishide, H., Uchiyama, I., Tatarazako, N. and Iguchi, T.	2014	microarray	Daphnia magna	methoprene; fenoxycarb; epofenonane
Molecular impact of propiconazole on Daphnia magna using a reproduction-related cDNA array	Soetaert, A., Moens, L. N., Van der Ven, K., Van Leemput, K., Naudts, B., Blust, R. and De Coen, W. M.	2006	microarray	Daphnia magna	propiconazole
Molecular mechanisms of tolerance to cyanobacterial protease inhibitors revealed by clonal differences in Daphnia magna	Schwarzenberger, A., Kuster, C. J. and Von Elert, E.	2012	qPCR	Daphnia magna	dietry trypsin inhibitor
Molecular responses during cadmium-induced stress in Daphnia magna: integration of differential gene expression with higher-level effects	Soetaert, A., Vandenbrouck, T., van der Ven, K., Maras, M., van Remortel, P., Blust, R. and De Coen, W. M.	2007	microarray	Daphnia magna	cadmium
Neverland regulates embryonic moltings through the regulation of ecdysteroid synthesis in the water flea Daphnia magna, and may thus act as a target for chemical disruption of molting	Sumiya, E., Ogino, Y., Toyota, K., Miyakawa, H., Miyagawa, S. and Iguchi, T.	2016	qPCR	Daphnia magna	ecdysteroids
Nickel and binary metal mixture responses in Daphnia magna: molecular fingerprints and (sub)organismal effects	Vandenbrouck, T., Soetaert, A., van der Ven, K., Blust, R. and De Coen, W.	2009	microarray	Daphnia magna	Nickel; Cadmium; Lead
Nickel response in function of temperature differences: effects at different levels of biological organization in Daphnia magna	Vandenbrouck, T., Dom, N., Novais, S., Soetaert, A., Ferreira, A. L., Loureiro, S., Soares, A. M. and De Coen, W.	2011	qPCR, microarray	Daphnia magna	nickel; temperature
NMDA receptor activation upstream of methyl farnesoate signaling for short day-induced male offspring production in the water flea, Daphnia pulex	Toyota, K., Miyakawa, H., Yamaguchi, K., Shigenobu, S., Ogino, Y., Tatarazako, N., Miyagawa, S. and Iguchi, T.	2015	RNA-Seq	Daphnia pulex	Methyl farnesoate

Phenotypic plasticity in three Daphnia genotypes in response to predator kairomone: evidence for an involvement of chitin deacetylases	Christjani, M., Fink, P. and von Elert, E.	2016	qPCR	Daphnia pulex	Fish Kairomones
Population genomics of resource exploitation: insights from gene expression profiles of two Daphnia ecotypes fed alternate resources	Dudycha, J. L., Brandon, C. S. and Deitz, K. C.	2012	microarray	Daphnia pulex	Ankistrodesmus falcatus; seston
Predator-induced defences in Daphnia pulex: Selection and evaluation of internal reference genes for gene expression studies with real-time PCR	Spanier, K. I., Leese, F., Mayer, C., Colbourne, J. K., Gilbert, D., Pfrender, M. E. and Tollrian, R.	2010	qPCR	Daphnia pulex	Fish kairomones
ProPhenolOxidase in Daphnia magna: cDNA sequencing and expression in relation to resistance to pathogens	Labbe, P. and Little, T. J.	2009	qPCR	Daphnia magna	Pasteuria ramosa
Quantification of differentially expressed genes in Daphnia magna exposed to rubber wastewater	Jo, H. J. and Jung, J.	2008	qPCR	Daphnia magna	Rubber wastewater
Regulation and dysregulation of vitellogenin mRNA accumulation in daphnids (Daphnia magna)	Hannas, B. R., Wang, Y. H., Thomson, S., Kwon, G., Li, H. and Leblanc, G. A.	2011	qPCR	Daphnia magna	Diethyl stilbestrol; 4-Nonyl phenol; Piperonyl butoxide; Cyproterone acetate; Kinoprene; Methoprene; Pyriproxyfen; Fenoxycarb; Bisphenol A; Fenarimol; Testosterone; Chloroform; Zinc (as sulfate); Pentachlorophenol; Methylene chloride; Naphthalene; Acetone; Caffeine; N; N-diethyl-meta-toluamide (DEET); Atrazine; Pyrene; Triclosan; Cadmium (as chloride); Fipronil; 20-Hydroxyecdysone; Chlordane; Dieldrin; Ponasterone A; Methyl farnesoate
Sequence Conservation and Sexually Dimorphic Expression of the Ftz-F1 Gene in the Crustacean Daphnia magna	Mohamad Ishak, N. S., Kato, Y., Matsuura, T. and Watanabe, H.	2016	qPCR	Daphnia pulex	Fenoxycarb
Silver Nanowire Exposure Results in Internalization and Toxicity to Daphnia magna	Scanlan, L. D., Reed, R. B., Loguinov, A. V., Antczak, P., Tagmount, A., Aloni, S., Nowinski, D. T., Luong, P., Tran, C., Karunaratne, N., Pham, D., Lin, X. X., Falciani, F., Higgins, C. P., Ranville, J. F., Vulpe, C. D. and Gilbert, B.	2013	microarray	Daphnia magna	Silver Nanowire
Synergistic interactions of biotic and abiotic environmental stressors on gene expression	Altshuler, I., McLeod, A. M., Colbourne, J. K., Yan, N. D. and Cristescu, M. E.	2015	qPCR	Daphnia pulex	calcium; fish kairomones; Light Dark cycle
Systems Biology Approach Reveals a Calcium-Dependent Mechanism for Basal Toxicity in Daphnia magna	Antczak, P., White, T. A., Giri, A., Michelangeli, F., Viant, M. R., Cronin, M. T., Vulpe, C. and Falciani, F.	2015	qPCR	Daphnia magna	cadmium; nickel; copper; selenium; zinc; manganese; arsenic;silver; chromium; pyriproxyfen;ponasterone A; methyl farnesoate;toxaphene; beta-estradiol;aroclor 1242;20-hydroxyecdysone;methoxychlor; nonylphenol; permethrin; bifenthrin; ?-cyhalothrin; diazinon; parathion; chlorpyrifos; toluene; phenol; beta-benzene hexachloride;dichlorobenzene; phenanthrene; atrazine; methyl tert-butyl ether; chloroform; acrylonitrile; bis(2-ethylhexyl)phthalate; trichloroethylene; 2-chloroethyl vinyl ether; thapsigargin
Systems biology meets stress ecology: linking molecular and organismal stress responses in Daphnia magna	Heckmann, L. H., Sibly, R. M., Connon, R., Hooper, H. L., Hutchinson, T. H., Maund, S. J., Hill, C. J., Bouetard, A. and Callaghan, A.	2008	qPCR, microarray	Daphnia magna	Ibuprofen
Target gene approaches: Gene expression in Daphnia magna exposed to predator-borne kairomones or to microcystin-producing and microcystin-free Microcystis aeruginosa	Schwarzenberger, A., Courts, C. and von Elert, E.	2009	qPCR	Daphnia magna	fish kairomones; microcystin
Temporal Expression of the Clock Genes in the Water Flea Daphnia pulex (Crustacea: Cladocera)	Bernatowicz, P. P., Kotwica-Rolinska, J., Joachimiak, E., Sikora, A., Polanska, M. A., Pijanowska, J. and Bebas, P.	2016	qPCR	Daphnia pulex	Light Dark Cycle
The evolution of salinity tolerance in Daphnia: a functional genomics approach	Latta, L. C., Weider, L. J., Colbourne, J. K. and Pfrender, M. E.	2012	microarray	Daphnia pulex	salinity
The good, the bad, and the toxic: approaching hormesis in Daphnia magna exposed to an energetic compound	Stanley, J. K., Perkins, E. J., Habib, T., Sims, J. G., Chappell, P., Escalon, B. L., Wilbanks, M. and Garcia-Reyero, N.	2013	microarray	Daphnia magna	Tri Nitro Toluene (TNT)
The potential of dietary polyunsaturated fatty acids to modulate eicosanoid synthesis and reproduction in Daphnia magna: a gene expression approach	Schlotz, N., Sorensen, J. G. and Martin-Creuzburg, D.	2012	qPCR	Daphnia magna	Different algae food
Tissue-specific expression of a bHLH-PAS protein homologous to ARNT during the development of crustacean Daphnia magna	Tokishita, S., Kimura, S., Mandokora, Y., Kato, K., Shiga, Y., Takahashi, Y., Ohta, T. and Yamagata, H.	2006	qPCR	Daphnia magna	hypoxia
Toxicogenomic responses of nanotoxicity in Daphnia magna exposed to silver nitrate and coated silver nanoparticles	Poynton, H. C., Lazorchak, J. M., Impellitteri, C. A., Blalock, B. J., Rogers, K., Allen, H. J., Loguinov, A., Heckman, J. L. and Govindasmawy, S.	2012	microarray	Daphnia magna	Silver nanoparticles
Transcriptional changes during Daphnia pulex development indicate that the maturation decision resembles a rate more than a threshold	Harney, E., Plaistow, S. J. and Paterson, S.	2015	microarray	Daphnia pulex	Developmental Stages
Transcriptional profiling of predator-induced phenotypic plasticity in Daphnia pulex	Rozenberg, A., Parida, M., Leese, F., Weiss, L. C., Tollrian, R. and Manak, J. R.	2015	RNA-Seq	Daphnia pulex	fish kairomones

Transcriptomic alterations in Daphnia magna embryos from mothers exposed to hypoxia	Lai, K. P., Li, J. W., Chan, C. Y., Chan, T. F., Yuen, K. W. and Chiu, J. M.	2016	RNA-Seq	Daphnia magna	hypoxia
De Novo Transcriptome Assembly and Sex-Biased Gene Expression in the Cyclical Parthenogenetic Daphnia galeata	Huylmans, A. K., Lopez Ezquerra, A., Parsch, J. and Cordellier, M.	2016	RNA-Seq	Daphnia galeata	sex biased
